# Developmentally-programmed cellular senescence is conserved and widespread in zebrafish

**DOI:** 10.18632/aging.103968

**Published:** 2020-09-29

**Authors:** Sabela Da Silva-Álvarez, Jorge Guerra-Varela, Daniel Sobrido-Cameán, Ana Quelle, Antón Barreiro-Iglesias, Laura Sánchez, Manuel Collado

**Affiliations:** 1 Laboratory of Stem Cells in Cancer and Aging, Health Research Institute of Santiago de Compostela (IDIS), Xerencia de Xestión Integrada de Santiago (XXIS/SERGAS), Santiago de Compostela, Spain; 2 Department of Zoology, Genetics and Physical Anthropology, School of Veterinary, Universidade de Santiago de Compostela, Lugo, Spain; 3 Geneaqua S.L., Lugo, Spain; 4 Department of Functional Biology, CIBUS, Faculty of Biology, Universidade de Santiago de Compostela, Santiago de Compostela, Spain

**Keywords:** cellular senescence, development, zebrafish

## Abstract

Cellular senescence is considered a stress response imposing a stable cell cycle arrest to restrict the growth of damaged cells. More recently however, cellular senescence was identified during mouse embryo development at particular structures during specific periods of time. This programmed cell senescence has been proposed to serve developmental and morphogenetic functions and to potentially represent an evolutionary origin of senescence. Cellular senescence has also been described to take place during bird (chick and quail) and amphibian (xenopus and axoltl) development. Fish however, have been described to show a very narrow and restricted pattern of developmental cell senescence. Here we carried out a detailed characterization of senescence during zebrafish development and found it to be conserved and widespread. Apart from yolk and cloaca, previously described structures, we also identified senescence in the developing central nervous system, intestine, liver, pronephric ducts, and crystalline. Interestingly, senescence at these developing structures disappeared upon treatment with senolytic compound ABT-263, supporting their senescent identity and opening the possibility of studying the contribution of this process to development. In summary, our findings extend the description of developmentally-programmed cell senescence to lower vertebrates contributing to the notion of the relevance of this process for embryo development.

## INTRODUCTION

Cellular senescence is a state of permanent cell cycle arrest imposed on cells upon a wide range of potentially dangerous stressors and serving as a potent tumour suppressor mechanism [1, 2]. Recently, several studies reported the occurrence of cellular senescence in developing tissues of mammals (mice and humans), birds (chick and quail) and amphibians (xenopus and axoltl) in non-pathological conditions raising the possibility of an evolutionary origin of senescence as a positive morphogenetic and tissue remodelling force operating during development [3–6]. In this sense, the presence of cellular senescence during the early development of zebrafish was also reported recently [7]. Based on the detection of senescence-associated β-galactosidase (SA–β–gal) activity, a well-established senescent cell marker [8], these authors reported the presence of SA–β–gal staining only in the yolk and in the developing intestine. This is a much more restricted pattern of SA–β–gal than those reported in rodents and amphibians, in which this staining is observed in a variety of developing tissues including the neural tube or the pronephros among others [9]. Previously, other authors had used SA–β–gal staining in zebrafish to carry out a genetic screen to identify genes regulating cellular senescence [10]. If one compares the images of SA–β–gal from both studies, there is a clear discrepancy in the intensity and on the variety of labelled structures, although the later study did not report a detailed inspection of their SA–β–gal stainings. Here, we carried out a careful examination of the occurrence of SA–β–gal staining in developing zebrafish and found it to be widespread in a number of developing structures.

## RESULTS

**Figure 1:**
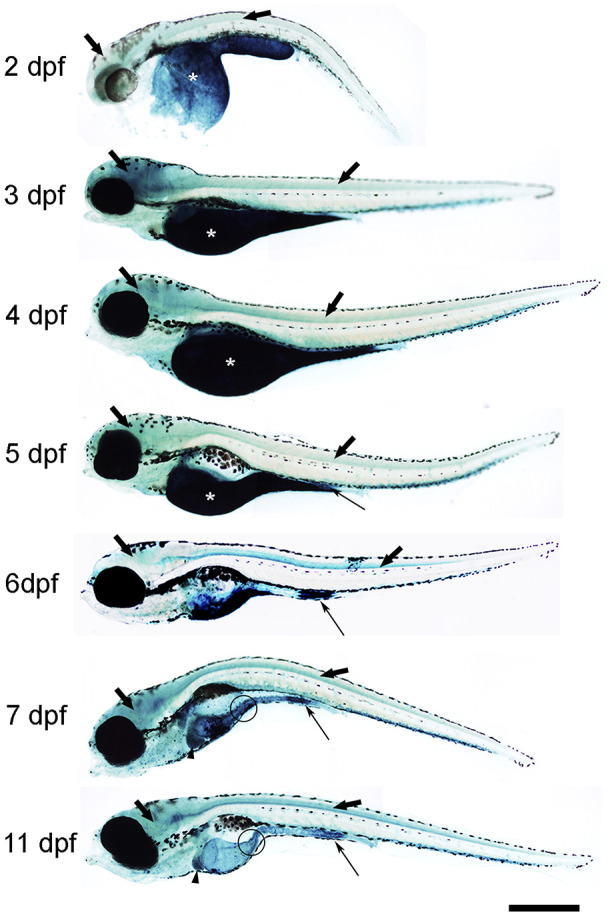
**Photomicrographs of whole-mounted developing zebrafish showing the presence of SA–β–gal staining.** Representative images of 2, 3, 4, 5, 6, 7 and 11 dpf zebrafish are shown. The asterisks indicate the presence of intense SA–β–gal staining in the yolk. Thin arrows indicate the presence of intense SA–β–gal staining in the caudal (cloacal) end of the intestine. Thick black arrows indicate the presence of SA–β–gal staining in the brain. Thick empty arrows indicate the presence of SA–β–gal staining in the spinal cord. Arrowheads indicate the presence of SA–β–gal staining in the liver. Circles indicate the presence of SA–β–gal staining in the oesophagus. Scale bar: 200 μm. Images are a composition of different pictures taken under the microscope overlapped together and modified using the same parameters.

**Figure 2:**
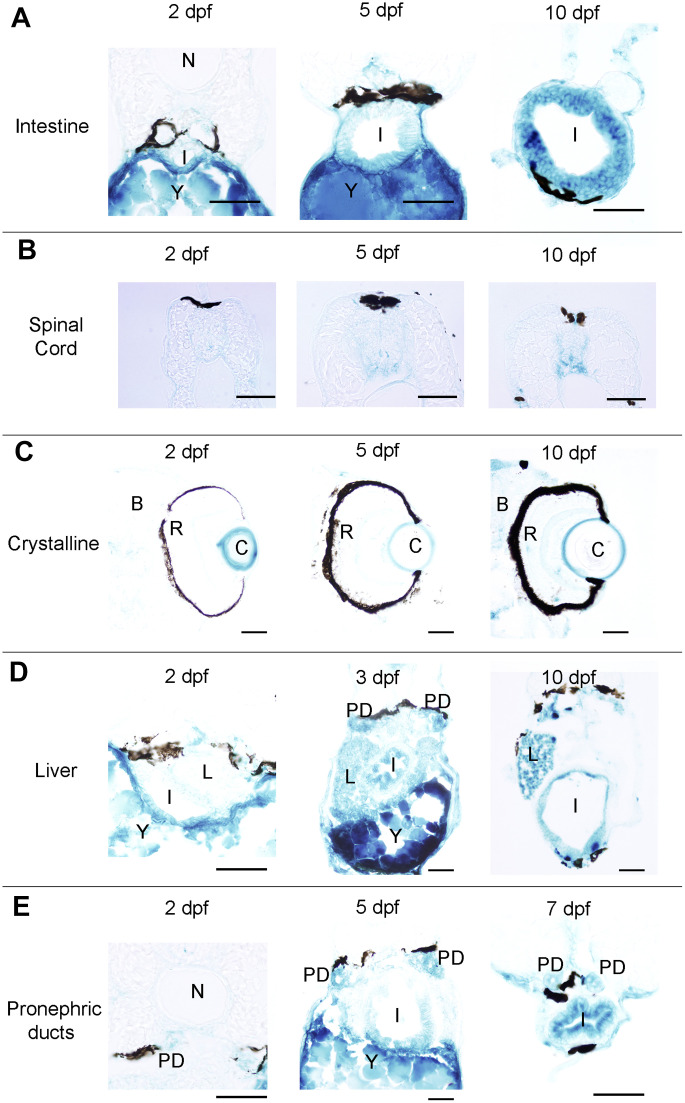
**Photomicrographs of transverse sections of developing zebrafish showing the presence of SA–β–gal staining in different organs.** (**A**) Photomicrographs showing the presence of SA–β–gal staining in the rostral part of the intestine. (**B**) Photomicrographs showing the presence of SA–β–gal staining in the spinal cord. (**C**) Photomicrographs showing the presence of strong SA–β–gal staining in the outer layer of the crystalline and weak SA–β–gal staining in the brain. (**D**) Photomicrographs showing the presence of SA–β–gal staining in the liver. Note that the intensity of staining in the liver is weaker in 2 dpf animals. (**E**) Photomicrographs showing the presence of SA–β–gal staining in the pronephric ducts. Note that the intensity of staining in the pronephric duct is weaker in 2 dpf animals. Dorsal is to the top in all sections. Abbreviations: B: brain, C: crystalline, I: intestine, N: notochord, L: liver, PD: pronephric ducts, R: retina, Y: yolk. Scale bars: 50 μm.

First, we analysed the spatio-temporal pattern of SA–β–gal staining in zebrafish from 2 to 11 days post-fertilization (dpf) (Figure 1). In whole-mounts, the intensity of SA–β–gal staining was weaker in 2 dpf animals and increased as development progressed, with the strongest intensity observed from 7 dpf onwards (Figure 1). In agreement with the results reported previously, we observed strong SA–β–gal staining in the yolk, a staining that disappeared as development progressed in concordance with the transient nature of this structure (Figure 1) [7]. SA–β–gal staining was also observed in the digestive system of developing zebrafish. As seen in whole-mount preparations from 5 dpf onwards, SA–β–gal staining was particularly strong in the caudal (cloacal) end of the intestine (Figure 1), which is also in agreement with the previous results [7], but the analysis of whole-mounts and transverse sections indicated that SA–β–gal staining is also present in the oesophagus (Figure 1) and the rostral intestine (Figure 1 and 2a) of developing zebrafish. The intensity of staining in the oesophagus and intestine was weaker in 2-3 dpf animals and increased with age (Figure 2A).

Interestingly, whole-mounts and transverse sections also revealed the presence of SA–β–gal staining in other structures that were not previously reported by Villiard and co-workers [7]. Whole-mount preparations showed clear SA–β–gal staining in the central nervous system (CNS) of developing zebrafish (Figure 1). This staining was weaker in 2 dpf animals and became progressively stronger as development progressed. In the spinal cord, SA–β–gal staining was stronger in the ventral portion of the cord and weaker in its dorsal portion (Figure 2B). This dorso-ventral difference in the spinal cord was clear from 5 dpf onwards (Figure 2B). Strong SA–β–gal staining was also observed in the outer layer of the crystalline lens of the eye, in the glomerulus and the caudally extending pronephric ducts, and in the liver. SA–β–gal staining was observed in the crystalline at all the analysed stages (Figure 2C), in the liver from 3-4 dpf onwards (Figure 2D), and in the glomerulus and pronephric ducts at all the analysed stages being weaker in 2 dpf embryos (Figure 2E). SA–β–gal staining in these structures might have been missed by previous authors that mainly used whole-mounted preparations to analyse cellular senescence in developing zebrafish. The difference in intensity of staining along development for various structures reinforces the idea of a transitory process taking place at precise developmental stages and disappearing after culminating its role. This is for example evident for the liver and cloaca at 11 dpf (Figure 1).

**Figure 3:**
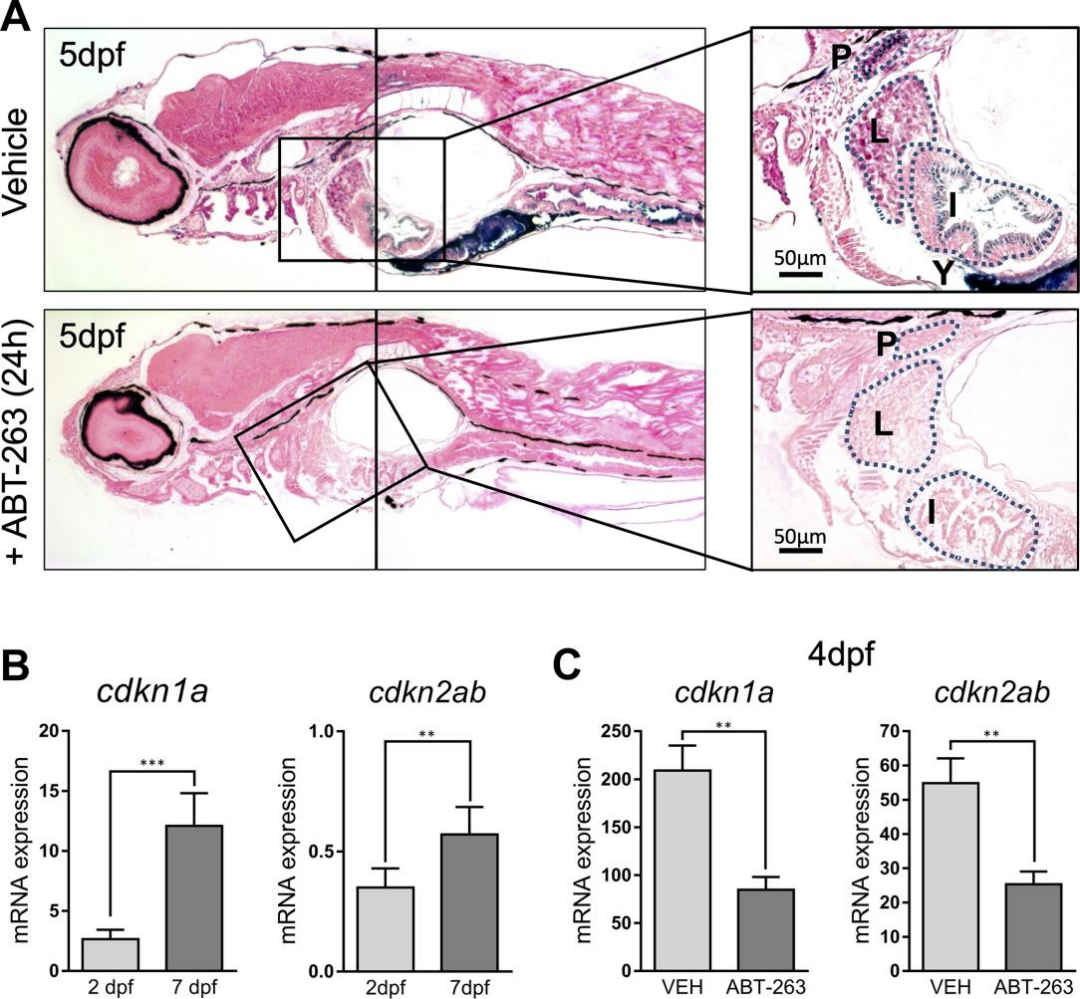
(**A**) Photomicrographs of whole-mounted developing zebrafish (5 dpf) stained for SA–β–gal in the absence (upper panels) or presence (lower panels) of senolytic compound ABT-263 for 24 h. Right panels show an amplified area showing a detail of positive structures. Scale bars: 50 μm. P: pronephric ducts; L: liver; I: intestine; Y: yolk. (**B**) Expression levels by QPCR of *cdkn1a* (left panel) and *cdkn2ab* (right panel) genes at 2 and 7 dpf relative to the housekeeping gene *rps11*. (**C**) Expression levels by QPCR of *cdkn1a* (left panel) and *cdkn2ab* (right panel) genes relative to the housekeeping gene *rps11* at 4 dpf after treatment with ABT-263 or vehicle (VEH) for 48 h. Data in (**B**) and (**C**) correspond to the average ± s.d. Statistical significance was assessed by the two-tailed Student’ s t-test: *** p < 0.001; ** p < 0.01. Samples were assessed in triplicates of pools of 30 larvae.

To rule out artefactual SA–β–gal staining, we decided to use a senolytic agent to remove senescent cells. Senolytics are compounds with a specific senescent cell killing activity. We treated 5 dpf larvae for 24h with ABT-263 (Navitoclax), an inhibitor of antiapoptotic proteins Bcl-2, Bcl-xL and Bcl-w with senolytic activity [11]. SA–β–gal staining of treated animals revealed a dramatically reduced staining along all the described structures (Figure 3A). In addition, we measured the whole embryo expression of *cdkn1a* and *cdkn2ab*, the genes coding for cell cycle regulators p21 and p16, respectively, known markers of cellular senescence. Levels of *cdkn1a* were clearly lower at 2 dpf, when SA–β–gal staining was weaker, compared to 7 dpf, a time at which SA–β–gal intensity was higher, nicely correlating with our stainings (Figure 3B). In contrast, ABT-263 treatment of 2 dpf embryos for 2 days prevented the increase in the expression of *cdkn1a* and *cdkn2ab* (another cell cycle inhibitor linked to senescence), reinforcing the idea of the existence of developmental senescence taking place in zebrafish embryos (Figure 3C).

## DISCUSSION

The identification of developmentally-programmed senescence in mouse embryos opened new perspectives on our understanding of this process. Senescence during embryo development has been observed in the context of organ regression, modulating cell population balance, and promoting tissue growth [12]. The observation of senescence operating on a context of tissue remodelling and sculpting structures in the embryo provides clues about a putative physiological role in the adult organism during tissue repair and in processes of regeneration, a possibility that has already been proved [13, 14]. It offers also a novel angle to consider the protumorigenic contribution of persistent senescence in line with the classical view of aberrant tissue repair signalling as the basis of cancer.

In this context, we wondered to which extent is this developmental senescence conserved in distant vertebrates, such as the zebrafish, a valuable animal model to study development. The initial description of senescence in zebrafish embryos revealed a very restricted and confined pattern of expression, although the analysis was not complete and relied on whole mount SA–β–gal stainings alone. We provide a detailed analysis of the structures showing SA–β–gal staining in developing zebrafish extending previous observations by other authors [7] to other tissues and organs, and reinforcing the relevance of developmentally-programmed cell senescence as a highly conserved phenomenon in vertebrates. The different fixing, staining and analytic conditions used in this study could explain the wider identification of developmental senescence. Still, the direct detection of senescence marker expression in the identified structures is lacking and could provide very valuable information. In addition, we show that senolytic treatment of embryos is effective removing these senescent cells, an untested possibility that offers now the opportunity to address directly the role of this process during embryogenesis.

These results, together with the identification of developmental senescence in amphibians, salamanders, birds and mammals, support the notion that cell senescence emerged early during the evolution of vertebrates to favour tissue remodelling and morphogenesis.

## MATERIALS AND METHODS

### Animals

Embryos were obtained from breeding of wild-type AB adult zebrafish. Animals were maintained in a controlled environment in a 14h light/10h dark cycle at 28°C [15]. The protocols used in this study were performed in compliance with the EU animal experimentation regulation (EU, 2010) and were approved by the Bioethics Committee for Animal Experimentation CEEA-LU (Universidade de Santiago de Compostela, Spain).

### Senescence-associated β-galactosidase activity

Determination of SA–β–gal activity was performed through the widely used X-gal staining method. Whole-mount stainings were performed essentially as originally described [8]. Briefly, larvae between 2 and 11 dpf (n=10 for each developmental time) were fixed for 20 min (room temperature) in 2% formaldehyde/0.2% glutaraldehyde, washed and incubated overnight at 37ºC with freshly made SA– β–gal solution: 1 mg of 5-bromo-4-chloro-3-indolyl beta-D-galactoside (X-Gal) per mL (Fisher Scientific), 40 mM citric acid/sodium phosphate pH 6.0, 5 mM K_3_Fe[CN]_6_, 5 mM K_4_Fe[CN]_6_, 150 mM NaCl, and 2 mM MgCl_2_. After staining, some larvae were washed with PBS, mounted with glycerol and photographed with an Olympus microscope. Other animals were rinsed in PBS, cryoprotected with 30% sucrose in PBS overnight at 4 ºC, embedded in Tissue Tek (Sakura, Torrance, CA, USA), frozen in liquid nitrogen-cooled isopentane and cut serially on a cryostat (16 μm sections) in the transverse plane. Sections were mounted on Superfrost ® Plus glass slides (Menzel, Braunschweig, Germany) using Mowiol ® (Sigma).

### ABT-263 treatment

ABT-263 (Navitoclax; a generous gift from Abbvie) was added directly to the water to a final concentration of 2 μg/mL for 24 h to 5 dpf larvae before fixation and SA–β–gal staining as above, or for 48 h for QPCR. Treated animals were embedded in paraffin for sectioning and further histological examination using classical eosin staining.

### Gene expression analysis

To measure RNA expression by quantitative RT-PCR, samples (pools of n=30; at 2, 4 or 7 dpf larvae) were disrupted using TissueLyser II and total RNA was extracted using the NucleoSpin® RNA kit (Macherey-Nagel) following the indications of the provider and DNAse treatment. After RNA quantification on nanodrop, the RNA was retrotranscribed into cDNA according to the manufacturer’s protocol (High-Capacity cDNA Reverse Transcription Kit, Applied Biosystems). Quantitative Real Time-PCR was performed using SYBR Green Power PCR Master Mix (Applied Biosystems) in an AriaMx real-time PCR system (Agilent technologies). Relative quantitative RNA was normalized using the housekeeping gene *rps11.* The primers used were: *rps11*-F: 5’-ACAGAAATGCCCCTTCACTG-3’; *rps11*-R: 5’-GCCTCTTCTCAAAACGGTTG-3’; *cdkn1a*-F: 5’-CGCAAACAGACCAACATCAC-3’; *cdkn1a*-R: 5’-ATGCAGCTCCAGACAGATGA-3’; *cdkn2ab*-F: 5’-CCGCACGGTGTCAATGAATC-3’; *cdkn2ab*-R: 5’-ATTTTCCCCCTCTCCAGGTG-3’.

### Statistical analysis

Samples were assessed in triplicates and statistical significance was assessed by the two-tailed Student’ s *t*-test: *** p < 0.001; ** p < 0.01.
